# Use of Primary Care Data for Detecting Impetigo Trends, United Kingdom, 1995–2010

**DOI:** 10.3201/eid1910.130433

**Published:** 2013-10

**Authors:** Laura J. Shallcross, Irene Petersen, Joe Rosenthal, Anne M. Johnson, Nick Freemantle, Andrew C. Hayward

**Affiliations:** University College London, London, UK

**Keywords:** impetigo, epidemiology, Staphylococcus aureus, primary care, United Kingdom, bacteria

## Abstract

Using a primary care database, we identified a major increase in impetigo in the United Kingdom during 1995–2010. Despite a doubled rate of primary care consultations, this increase was not identified by routine surveillance. Primary care databases are a valuable and underused source of surveillance data on infectious diseases.

Impetigo is a common, superficial bacterial infection primarily caused by *Staphylococcus aureus*. It is the fourth most common dermatologic condition among children seen in general practice; although most infections are mild, outbreaks have a considerable negative effect because infected children may be barred from attending schools and nurseries (*1*).

In the United Kingdom, hospitalizations for impetigo increased 5-fold from 1989–1990 through 2003–2004; among children, the increase was 12-fold (*2*). To investigate whether this increase was fueled by increasing rates of infection in the community rather than increased pathogenicity, we used a large and nationally representative primary care database to calculate time trends in incidence of consultation and medications prescribed for impetigo.

## The Study

We examined electronic patient records from The Health Improvement Network (THIN) database, a source of detailed clinical information about patient primary care consultations (*3*). In the United Kingdom, 98% of the population is registered with a general practitioner (a primary care physician who provides advice, treatment, and prescriptions and acts as a gatekeeper to specialist services) (*4*). Participating practices enter demographic and clinical data into the practice database by using Vision software (www.inps4.co.uk) every time a consultation takes place, generating a longitudinal medical record. Since 1990, symptoms, diagnoses, treatments, and referrals have been recorded by use of a hierarchical system of >103,000 Read codes (*5*). Prescriptions are recorded by using Multilex (www.fdbhealth.co.uk/solutions/multilex) drug codes, which link each drug formulation to the British National Formulary, a compendium of drugs arranged by system into 15 chapters. THIN contains the medical records of >3.7 million current patients (*3*) and is broadly representative of the UK population (*6*). Prescription and consultation rates in the dataset are comparable to those recorded by national statistics and external data sources (*7*,*8*). Adequacy of death data recording is assessed by identifying the date at which mortality rates recorded by the practice correspond to the national age and sex standardized mortality rate, defined as the acceptable mortality recording date. We included data from patients from practices that met acceptable mortality recording criteria and were fully computerized (*9*). Persons were eligible for study inclusion if they were registered with a participating practice from January 1, 1995, through December 31, 2010.

Read code lists were developed to identify patients seeking care for impetigo. A drug code list was developed to identify patients for whom topical fusidic acid (with or without a topical steroid) was prescribed, based on the relevant chapter of the British National Formulary.

To assess time trends, we included the first consultation for impetigo for patients 0–14 years of age or the first prescription of fusidic acid; that is, patients were counted once per practice. Patients left the study on the earliest of the following dates: date of consultation or prescription, date of leaving the practice, date of death, or date the study ended (December 31, 2010). We used Poisson regression to calculate the incidence of first consultations or prescriptions per year. The denominator was the total number of person-years contributed by patients in the sample population for each corresponding calendar year.

The THIN program of anonymized data provision for researchers was approved by the National Health Service South East Multi-Centre Research Ethics Committee in 2002. This study was approved by the THIN scientific review committee, reference 11–504.

**Figure 1:**
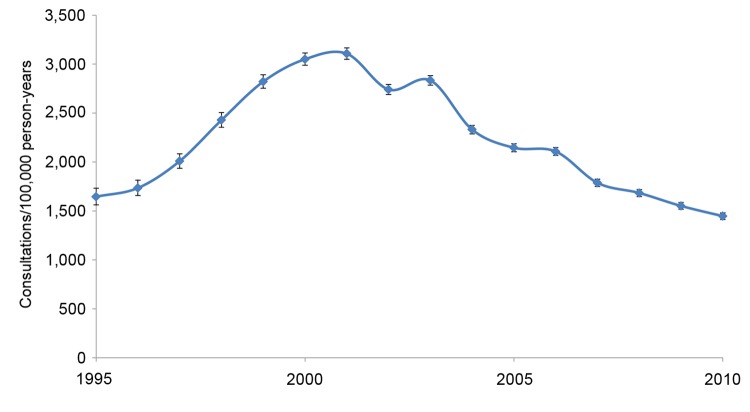
Rates of general practitioner consultation for impetigo among children 0–14 years of age, United Kingdom, 1995–2010. Error bars indicate 95% CIs.

**Figure 2:**
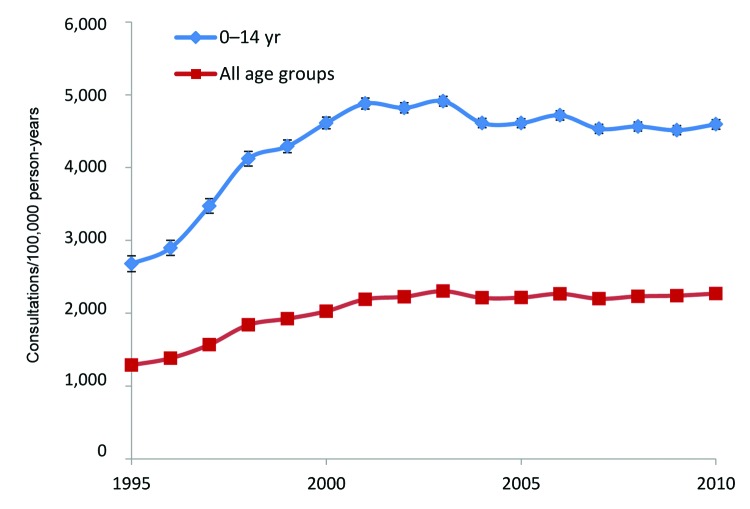
Rates of fusidic acid prescription by general practitioners, United Kingdom, 1995–2010. Error bars indicate 95% CIs.

During 1995–2010, a total of 130,095 children 0–14 years of age were seen by a general practitioner for impetigo. The annual incidence of infection increased from 1,646 (95% CI 1,561–1,733) consultations per 100,000 person-years in 1995 to 3,106 (95% CI 3,048–3,165) per 100,000 person-years in 2001 (Figure 1) and declined thereafter to 1,447 (95% CI 1,413–1,481) per 100,000 person-years in 2010. The incidence of fusidic acid prescription for the total population increased from 1,287 (95% CI 1,256–1,318) prescriptions per 100,000 person-years in 1995 to 2,308 (95% CI 2,289–2,326) per 100,000 person-years in 2003 and stabilized thereafter (Figure 2). Prescriptions were most frequently issued for children 0–14 years of age; incidence peaked in 2003 at 4,911 (95% CI 4,843–4,980) prescriptions per 100,000 person-years. At the peak of the epidemic, there were ≈130,000 more general practitioner consultations for impetigo; this estimate is based on an estimated population of 9,375,100 children <15 years of age in England in 1995 and 9,282,700 in 2001 (*10*).

## Conclusions

A major increase in impetigo in children in the United Kingdom was not detected by routine surveillance data. The increased number of infections placed a substantial burden on primary care, adding 130,000 consultations in England at the peak of transmission. In England and Wales, the Royal College of General Practitioners Research and Surveillance Centre network of ≈100 sentinel practices is responsible for alerting general practitioners to major trends in the incidence of common conditions, such as influenza and impetigo, by providing weekly reports of disease incidence (*11*). Although this system detected a comparable increase in the rate of consultations for impetigo, the data were not used to alert general practitioners about a major increase in impetigo in the community because the system is designed to detect rapid changes in incidence, such as occur during an influenza epidemic.

Over the past decade, several European countries have reported a rise in general practice consultations for impetigo (*12*,*13*). Many European countries have established primary care databases, such as the Information System for the Development of Research in Primary Care in Catalonia (*14*) or the Health Search Database in Italy (*15*). Although these databases are smaller than the THIN database (*3*), they are sufficiently large and well-established to be used to examine national consultation and prescription trends in infectious diseases, offering the potential for a powerful international infectious disease surveillance network.

This study’s strengths lie in its scale and the fact that the database is nationally representative, containing the medical records of ≈6% of the UK population (*4*). The study’s limitations lie in the fact that the database was designed for patient management, not research. We acknowledge that comparing population rates for consultation and prescriptions might not accurately represent the association between prescriptions and disease at an individual level. Patients seeking consultation for impetigo were identified by diagnostic Read codes, and some general practitioners might prescribe fusidic acid without recording a diagnostic code. Identifying patients by Read code alone might underestimate incidence, whereas identifying patients by prescription data might lead to an overestimation because drugs are not always prescribed for a single condition. The actual incidence of impetigo in the community might have been higher because not all persons with impetigo would have consulted a general practitioner.

Although changes in health-seeking behavior or data recording could underlie the increased consultation rate, the fact that similar increases were reported from other primary and secondary care datasets suggests that our findings are not artifacts. The Read codes used to identify patients were unchanged throughout the study period, and we are unaware of any changes in clinical practice that would lead to an increased tendency to diagnose impetigo.

Impetigo is frequently dismissed as a mild infection that spontaneously resolves with a good outcome (*1*). By contrast, this study suggests that an undetected increase in impetigo in the community drove a major increase in hospital admissions of children in England from 1989–1990 through 2003–2004. Awareness of this epidemic by general practitioners could have triggered development of specific guidelines on the management of this condition, potentially improving treatment outcomes and reducing hospital admissions. Routinely collected primary care data are an underused and potentially rich source of information about infectious diseases in the community. We should do more to find novel ways of incorporating this information into international surveillance networks and using it to guide evidence-based treatment and prescribing decisions in primary care.
